# Significance of Glomerular Immune Reactivity in Time Zero Biopsies for Allograft Survival Beyond IgA

**DOI:** 10.3389/fmed.2021.656840

**Published:** 2021-04-06

**Authors:** Eva Vonbrunn, Sofya Serikova, Christoph Daniel, Kerstin Amann, Johannes Schödel, Björn Buchholz, Fulvia Ferrazzi, Katharina Heller, Hendrik Apel, Maike Büttner-Herold

**Affiliations:** ^1^Department of Nephropathology, Institute of Pathology, Friedrich-Alexander-University Erlangen-Nuremberg and University Hospital, Erlangen, Germany; ^2^Department of Nephrology and Hypertension, Friedrich-Alexander-University Erlangen-Nuremberg and University Hospital, Erlangen, Germany; ^3^Department of General Pathology and Pathological Anatomy, Institute of Pathology, Friedrich-Alexander-University Erlangen-Nuremberg and University Hospital, Erlangen, Germany; ^4^Department of Urology and Paediatric Urology, Friedrich-Alexander-University Erlangen-Nuremberg and University Hospital, Erlangen, Germany

**Keywords:** complement, renal tranplantation, time zero biopsy, transplant quality, immune reactivity

## Abstract

The quality of a renal transplant can influence the clinical course after transplantation. Glomerular immune reactivity in renal transplants has previously been described, focusing particularly on IgA, and has been shown to disappear in most cases without affecting the outcome. Here, we describe a cohort of time zero biopsies with regard to glomerular immune reactivity and implications for histomorphology and follow-up. 204 Time zero biopsies were analyzed by immunohistochemistry for glomerular immune reactivity. Time zero and 1-year biopsies were evaluated for histomorphological changes, which, together with clinical and follow-up data, were assessed for associations with glomerular immune profiles. Nearly half of the analyzed time zero biopsies showed glomerular immune reactivity with mesangial C3 being the most common (32.9%), followed by IgA (13.7%) and fullhouse patterns (6.9%). Strong C3 deposits (C3high) were only observed in deceased transplants. In the majority of cases immune reactivity was undetectable in follow-up biopsies and had no adverse effect on transplant function in follow-up of 5 years. In kidney pairs transplanted to different recipients a strong concordance of immune profiles in both kidneys was observed. Moreover, an association of male donor sex and deceased donor transplantation with the presence of immune reactivity was observed. In conclusion, glomerular immune reactivity is a very frequent finding in time zero biopsies, which seems to be determined by donor parameters including male sex and deceased donor transplants. It had no adverse impact on transplant function in 5-year follow-up. Glomerular immune reactivity in time zero biopsies, therefore, does not appear to indicate an inferior quality of the transplant.

## Introduction

Time zero biopsies offer the possibility to assess donor kidneys at the time of transplantation, which can help to interpret findings in later biopsies. In order to interpret the significance of time zero biopsy findings for the fate of the transplant it is important to correlate these findings with donor parameters and future transplant function.

Besides structural changes, a variety of glomerular immune reactivities have been described in time zero biopsies of donor kidneys in the past. In several series of time zero biopsies the incidence of mesangial IgA deposits was reported to be between 6.9 and 32.1% (1–8). This IgA reactivity gradually disappeared in most cases (4, 6–8) without influencing graft survival (4, 8) and function (6, 8). Glomerular IgM and C3 (7, 8) and less frequently IgG (3, 8) were also reported to vanish in the majority of cases in follow-up biopsies without adverse effects (7). Whereas, the vast majority of previous reports have been focusing on glomerular IgA (1–6, 8), in the present report we analyzed and compared different immune profiles in time zero biopsies including immunohistochemical fullhouse patterns and C3 reactivity as potential markers of complement activation in the transplanted organs.

Here, we analyzed a European cohort of time zero biopsies for the presence of glomerular immune reactivity and subdivided staining patterns into subgroups, including IgA, fullhouse, C3high, C3low and cases without significant immune reactivity. We compared morphologic, clinical and follow-up data between the groups.

## Materials and Methods

### Patients

**Figure 1:**
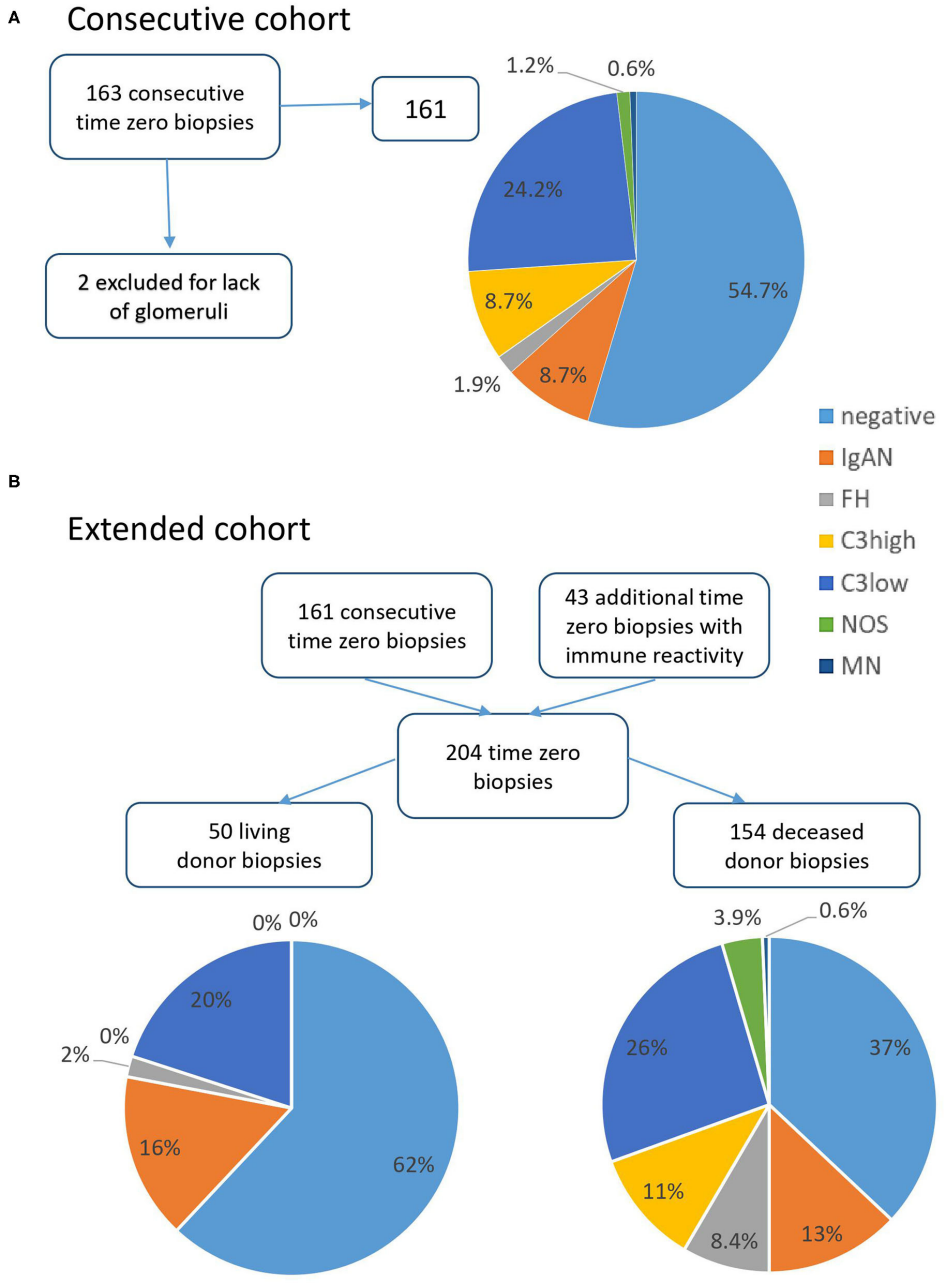
Relative distribution of immune pattern in time zero biopsies. **(A)** In the consecutive cohort 161 time zero biopsies were analyzed after exclusion of two cases with insufficient material. Over half of the cases showed no significant immune reactivity, about a quarter of cases mild C3 deposits (C3low) and the remainder different immune patterns including IgA, fullhouse (FH), and C3high. **(B)** Comparing living and deceased donor biopsies in the total cohort of 204 time zero specimens, negative cases were more prevalent in living donor organs and C3high staining was only observed in deceased donor transplants.

Consecutive and extended cohort: A total of 204 time zero specimens (203 kidney biopsies and one small kidney resection) of 191 donors, performed at the University Hospital of Erlangen and submitted to the Dept. of Nephropathology, were included. To assess the prevalence of glomerular immune reactivity we considered a consecutive cohort of 163 time zero biopsies performed between 06/2011 and 05/2013. Two cases were excluded for the lack of glomeruli, so that 161 cases were analyzed (Figure 1). As numbers of cases with immune reactivity were low in the consecutive cohort, for further analyses we randomly selected 43 additional time zero biopsies from 2010 to 2016, taken before or after the above-mentioned time span. These showed one of the below-defined glomerular immune patterns, in order to improve representativeness and comparability, for a total of 204 analyzed biopsies. The use of archived renal specimens was approved by the local Ethics committee (reference number 4415). Donor parameters included: sex, age, living/deceased donor, creatinine (mg/dl) and glomerular filtration rate (GFR; ml/min) before transplantation, body-mass index (BMI), cold and warm ischemia time (minutes), presence/absence of proteinuria, of a history of smoking, of diabetes mellitus, and arterial hypertension. Recipient parameters included sex, age, BMI; native kidney disease, number of hemodialyses required after transplantation, renal transplant (Rtx) and patient survival (yes/no), post-transplant renal function (primary/delayed/no function, organ loss or death), follow-up creatinine and GFR at 1 to 5 years post transplantation. GFR was calculated according to the CKD-EPI formula as described by Levely et al. (9) using patient serum creatinine and age under specification of race, sex and serum creatinine level. To achieve better comparability children <16 years were excluded from statistical analyses of creatinine and GFR.

### Immunohistochemistry

Immunohistochemical stainings with antibodies specific for IgA, IgG, IgM, C1q, C3c (all polyclonal: Cat. No. IgA A0262, IgG A0423, RRID:AB_2335700; IgM A04202, RRID:AB_578520; C1q A013602, RRID:AB_578496; C3c A006202, RRID:AB_578477, Agilent, Santa Clara, CA, USA) were performed on formalin fixed and paraffin-embedded (FFPE, 1μm sections) material with current standard methods after digestion with protease from Streptomyces griseus (Sigma-Aldrich, Munich, Germany, P5147) on a Ventana Benchmark stainer (Roche, Basel, Switzerland) or manually before 2011. Limited staining in the glomerular vascular pole was scored as negative. The intensity of staining was categorized into 4 grades: grade 0 (none), grade 1 (mild), grade 2 (moderate), grade 3 (strong).

In case of IgA reactivity in the time zero biopsy additional stainings for C4d (polyclonal, rabbit anti-C4d, 1:500, Cat. No. RBK061, RRID:AB_2864450, Zytomed Systems GmbH, Bargteheide, Germany; antigen-retrieval with ULTRA CC1 buffer, Roche) and galactose-deficient IgA (rat anti-Gd-IgA1, clone KM55, 1:100, Cat. No. 10777, Immuno-Biological Laboratories, Minneapolis, MA, USA; antigen-retrieval with protease digestion) were performed manually.

### Study Groups

According to the immunohistochemical findings biopsies were divided into the following subgroups: (i) negative: no glomerular immune reactivity except IgM and/or C1q, (ii) IgA: IgA deposits without fullhouse pattern, (iii) FH: fullhouse pattern with positivity of IgA, IgG, IgM, C1q and C3, (iv) C3high: C3 2+/3+ without IgA, IgG or C1q, (v) C3low: C3 1+ without IgA, IgG or C1q, (vi) MN: membranous pattern, (vii) NOS (not otherwise specified): mesangial immune reactivity exceeding the definition of negative, not meeting criteria of any other pattern.

### Follow-Up Biopsies

A total of 176 follow-up biopsies were evaluated, which were taken 4-1227 days post transplantation. These included 1-year biopsies (*n* = 111) and follow-up biopsies at other points in time (*n* = 65). Clinical indications for renal biopsy as retrieved from the accompanying files in the total of 176 follow-up biopsies were protocol biopsy (3 months or 1-year) in 96, rise in creatinine in 45 (one with simultaneous proteinuria), delayed graft function in 15, positive polyomavirus serology in seven, proteinuria in four, donor-specific antibodies in six, suspicion of rejection in two and not reported in one. Main diagnoses in the original reports included no rejection/significant pathology in 57, at least moderate acute tubular injury in 33, Borderline changes in 24, T-cell mediated rejection (TCMR) in 20, antibody-mediated rejection (ABMR) in 4 and suspicion of ABMR in 2, combined TCMR/ABMR or Borderline/ABMR in 2, polyomavirus-nephropathy in 10 (one accompanied by TCMR), interstitial fibrosis and tubular atrophy of ≥ 20% as main finding in 12, glomerulonephritis in eight deriving from 5 patients and interpreted as recurrence in three patients (glomerulonephritis was accompanied by acute tubular injury in three, by Borderline changes in two and by vascular hyaline microthrombi in one biopsy), cholesterol embolism in one, thrombotic microangiopathy in one, ischemic infarction in one and findings were insufficient for a diagnosis in one.

For all cases included in the cohort of time zero biopsies the respective 1-year biopsies were evaluated whenever available (*n* = 111) including all 12-months protocol biopsies and, in cases without protocol biopsy, any other biopsy taken between 9 and 15 months after transplantation, whenever available. Additionally, in IgA (22/28), FH (12/14), C3high (16/17), C3low (24/50), NOS (4/6) and MN (1/1) first follow-up biopsies after transplantation (irrespective of the time of biopsy) were assessed by immunohistochemistry (markers positive in the time zero biopsy were re-analyzed) to evaluate for persistence of glomerular immune reactivity. Immunohistochemistry was performed in 79 first follow-up biopsies, of which 16 were 1-year biopsies. In cases with persistence of glomerular immune reactivity in 1st follow-up 1-year biopsies or other available biopsies were analyzed by immunohistochemistry until negative or no further biopsy was available. A 2nd follow-up biopsy after persistence was evaluated in seven cases (6 1-year biopsies) and a 3rd biopsy in one case.

### Histological Evaluation of Time Zero Biopsies and 1-Year Biopsies

In time zero and 1-year biopsies total and globally sclerosed glomeruli were counted, interstitial fibrosis and tubular atrophy (IFTA) was estimated in steps of 5%, arteriosclerosis was scored as described previously (10). Matrix expansion (mesangial matrix >2 mesangial cell nuclei) was assessed as present or absent. In time zero biopsies, additionally, the degree of acute tubular injury (0: no/minimal, 1 <25%, 2:25–49%, 3:50–74%, 4 ≥ 75% of tubules involved) was scored.

### Statistical Analyses

Statistical analyses were performed using IBM SPSS Version 24. For the comparison of ordinal and numerical variables between either two or more independent groups Mann-Whitney and Kruskal-Wallis tests were used, respectively. Bonferroni correction was applied after *post-hoc* testing for Kruskal-Wallis. To test the association between nominal variables Pearson's chi-square test was used or alternatively Fisher's exact test when expected values were <5. Results with *p*-values < 0.05 were considered statistically significant. *Post-hoc* analysis for Pearson's chi-square test was performed using the standardized residuals (11) and correcting *p*-values using Bonferroni.

## Results

### Recipient and Donor Characteristics

Recipients were 50.1 years old (mean, standard deviation (SD) 16) including 131 men and 73 women. Native kidney disease in the clinical files included IgA in 18 cases, other glomerulonephritis (GN) in 31, hypertensive nephropathy (NP) in 25, autosomal dominant polycystic kidney disease in 28, diabetic NP in 12, congenital renal dysplasia in 10, vesico-ureteral reflux in five, amyloidosis and focal-segmental glomerulosclerosis each in three, interstitial nephritis in two and hemolytic uremic syndrome, nephronophthisis, calcineurin-inhibitor toxicity and familial mediteranean fever in one each and was unknown in 62 patients.

The mean age of the donors was 51.1 years (SD 15.4) including 88 men and 103 women. 67/184 donors were smokers, 11/177 were diabetic, 56/178 had a history of arterial hypertension and 43/182 were proteinuric. 154 (75.5%) transplant kidneys derived from deceased (mean age 50.5 years, SD 17.5; 69 men/72 women; 57/136 smokers; 11/128 diabetic; 49/129 hypertensive, 43/135 proteinuric) and 50 (24.5%) from living donors (mean age 52.7 years, SD 6.5; 19 men/31 women; 10/48 smokers; 0/49 diabetic; 7/49 hypertensive, 0/47 proteinuric).

### Composition of the Consecutive Cohort

Of 163 consecutive time zero biopsies performed between 06/2011 and 05/2013 161 were included for further analyses to estimate the prevalence of different immune patterns (Figure 1A). In this cohort, 88 biopsies (54.7%) showed no immune reactivity (negative), in 14 biopsies (8.7%) IgA was found, in 3 a fullhouse pattern (FH, 1.9%), in 14 (8.7%) moderate to strong C3 reactivity (C3high) and in 39 (24.2%) mild C3 positivity (C3low). In two (1.2%) biopsies a mesangial immune pattern was present not fitting one of the before described patterns (NOS, not otherwise specified) and in 1 biopsy (0.6%) positivity in a membranous pattern (MN) was present.

### Extended Cohort and Persistence

In order to increase group sizes for further statistical analyses and for better comparability 43 additional cases with glomerular immune reactivity in the time zero biopsy were added, making a total of 204 cases (Figure 1B). Comparing the distribution of immune patterns between living and deceased donor specimens (Figure 1B), in 50 transplants from living donors 31 (62%) showed negative immunohistochemistry and none of the cases showed C3high reactivity as opposed to transplants of deceased donors, which showed negative immunohistochemistry in only 57/154 specimens (37%, *p* = 0.002) and C3high in 17 cases (11%, *p* = 0.015). In the other groups no significant differences between living and deceased donors were observed (all *p* > 0.05).

**Figure 2:**
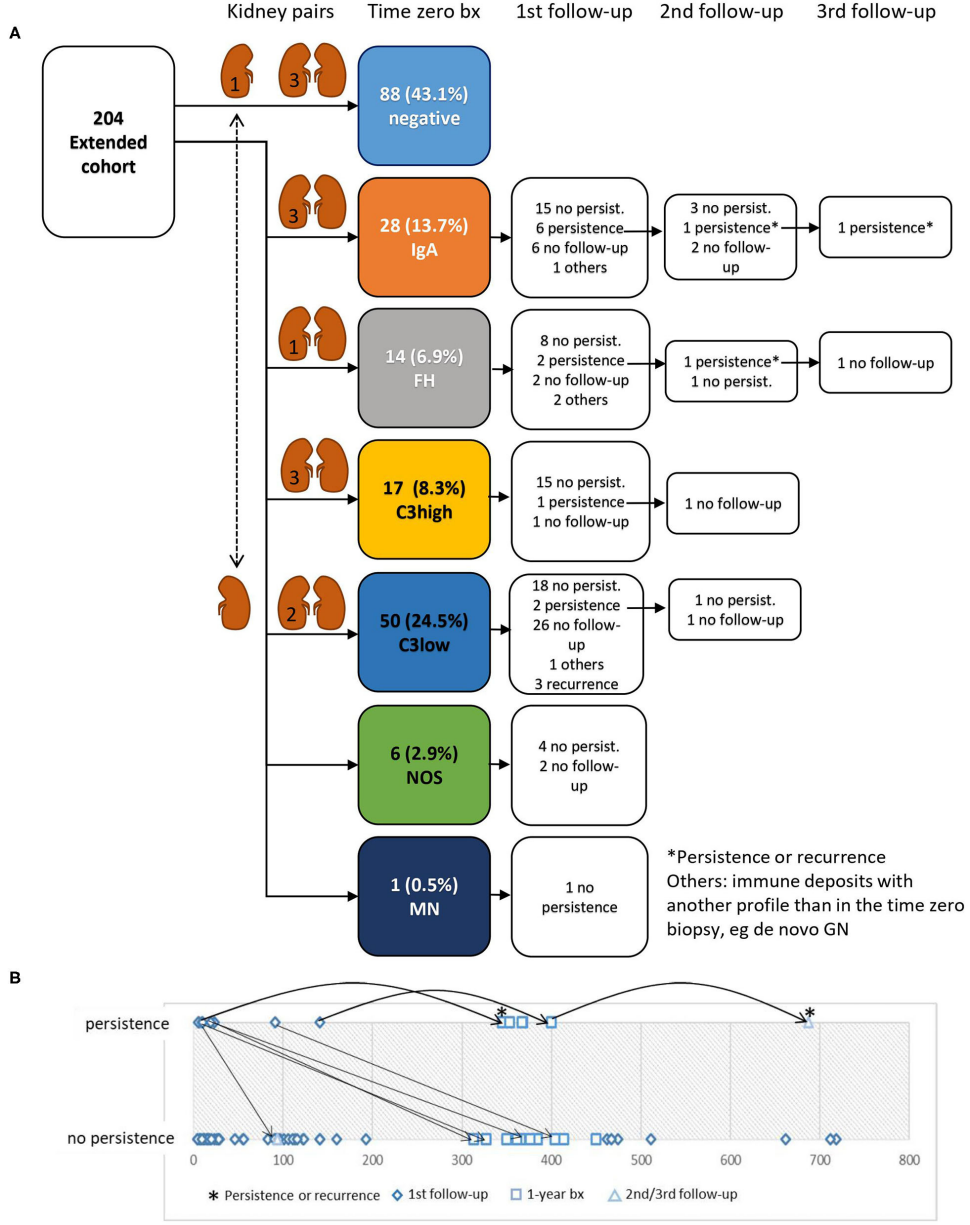
Disappearance and persistence of immune reactivity in follow-up biopsies. **(A)** Of 204 specimens included in the analysis 116 showed glomerular immune reactivity exceeding the definition of negative. In 79 cases immunohistochemical follow-up analyses were performed. In the majority of cases immune reactivity had already vanished in the 1st follow-up biopsy. In two cases with persistence (asterisks) it was unclear whether immunoreactivity was due to persistence or a recurrence of the native kidney disease. The cohort included 13 kidney pairs transplanted to different recipients, which in 12 cases showed the same immune profile (indicated by pictograms of kidney pairs and the number of cases in the left kidney). In one kidney pair one biopsy showed a C3low pattern and the corresponding kidney biopsy was negative for immune reactivity. **(B)** Time course of persistence of glomerular immune reactivity. In the majority of 1st follow-up biopsies (diamond) and 1-year biopsies (square) no persistence was observed from the beginning. In four of the biopsies with persistence and first follow-up shortly after transplantation (<100 days) immune reactivity vanished in the 2nd or 1-year follow-up biopsies. The two cases with persistence even in the 2nd or 3rd follow-up (asterisks) were possible recurrences of the native kidney disease.

In total, in the extended cohort 88 negative cases, 28 IgA, 14 FH, 17 C3high, 50 C3low, six immune reactivity NOS and 1 MN were included (Figure 2). Immunohistochemical findings are shown in Table 1. Isolated mesangial IgM reactivity (sometimes accompanied by C1q) was present in the vast majority of biopsies (79.4%) and was interpreted as unspecific deposition. IgM was associated with higher glomerular filtration rates and lower creatinine values of the donors prior to explantation compared to cases without glomerular IgM (*p* = 0.017 and 0.035 in all biopsies and *p* = 0.028 and 0.036 assessing the negative group only, data not shown).

**Table 1:** Immunohistochemical findings in time zero biopsies.

	**All**	**Negative**	**IgA**	**FH**	**C3high**	**C3low**	**NOS**	**MN**
IgA	42/204, 20.6%	0/88	28/28	14/14	0/17	0/50	0/6	0/1
IgG	23/204, 11.3%	0/88	5/28	14/14	0/17	0/50	3/6	1/1
IgM	162/204, 79.4%	66/88	25/28	14/14	12/17	39/50	5/6	1/1
C1q	30/204, 14.7%	8/88	2/28	14/14	0/17	0/50	6/6	0/1
C3	112/204, 54.9%	0/88	25/28	14/14	17/17	50/50	6/6	0/1
C4d	n.d.	n.d.	2/24	8/12	n.d.	n.d.	n.d.	n.d.
KM55	n.d.	n.d.	1/27	0/12	n.d.	n.d.	n.d.	n.d.

*Data are shown as number of positive cases/number of total cases, percentage. n.d., not done*.

The cohort included 13 kidney pairs, which were transplanted to different recipients. In 12 of them the immune profile in the time zero biopsy was the same in both organs including one case with fullhouse, 3 with IgA, 3 with C3high, 2 with C3low reactivity and three negative for glomerular immune reactivity. In one case mild mesangial C3 reactivity (C3low) was seen in one kidney, but not in the other organ, which was negative in immunohistochemistry (Figure 2A).

**Figure 3:**
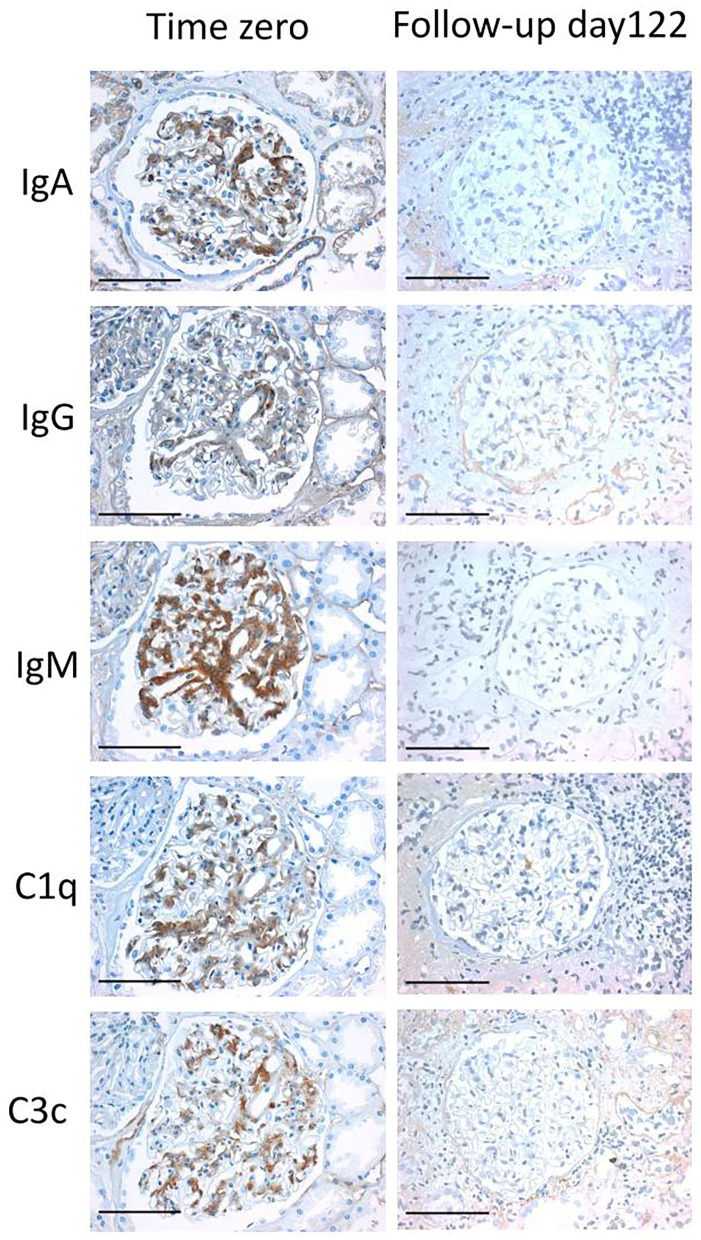
Fullhouse pattern disappearing in the follow-up biopsy. An example of an immunohistochemical fullhouse pattern is depicted in a time zero biopsy (left column), which has completely disappeared in the follow-up biopsy at day 122 (right column). All light microscopic pictures were taken with an AxioCam MRc and an Imager.A1 Axio microscope (Zeiss, Germany) at an original magnification of 400x. Scale bars indicate 100 μm.

Immunohistochemical follow-up was performed in 79 cases (Figure 2A) with immune reactivity in time zero biopsies. Immunohistochemistry turned negative in 65 cases (Figure 3), in four cases another immune profile than observed in the time zero biopsy developed and in three cases recurrence of the native kidney disease, as documented in the clinical files, was observed. In seven cases the findings persisted without further follow-up available. One of these cases showed again IgA reactivity and had IgA nephropathy as native kidney disease and one showed a fullhouse pattern and had a diagnosis of systemic lupus erythematosus. Hence, in these two cases a recurrence of the native kidney disease was a possible differential diagnosis of persistence of the initial findings observed in the time zero biopsy.

Having a glance at the time-course of persistence of immune deposits one can appreciate that persistence was much more frequent in the period of time shortly after transplantation and became more infrequent at later points in time (Figure 2B).

In 6 follow-up biopsies with persistent immune reactivity electron microscopy was performed, to evaluate for the ultrastructural correlate of immunohistochemical findings. In two biopsies definite osmiophilic deposits were observed (Supplementary Figure 1), in two questionable traces of osmiophilic deposits and in two no deposits could be appreciated.

### Clinical and Morphological Findings in Time Zero Biopsies With Glomerular Immune Reactivity

**Figure 4:**
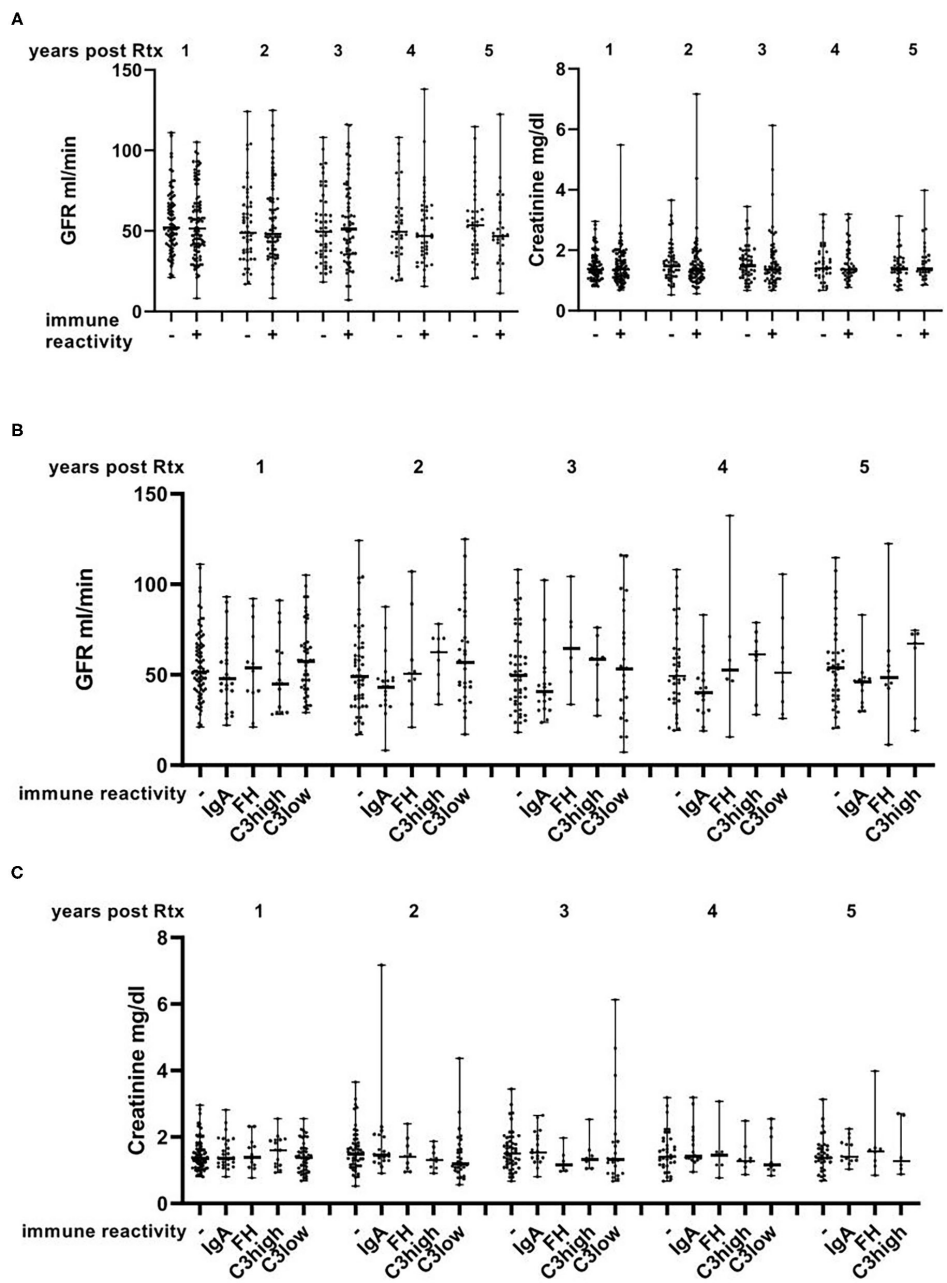
Course of GFR and creatinine in the first 5 years after transplantation. No differences with regard to GFR and creatinine were observed between cases that were positive and negative for immune reactivity **(A)** or across the different assessed subgroups **(B,C)** and no apparent overall deterioration of the renal function. Data are shown with median and range.

When comparing controls without immune reactivity with cases with IgA, FH, C3high or C3low no significant differences with regard to histomorphological parameters in time zero and 1-year biopsies were observed (Supplementary Table 1). Female donors were more common in the negative group (*p* = 0.002), and proteinuria in the donor was significantly more common in the C3high group (*p* = 0.002) compared to the remainder of analyzed groups. Donor age, creatinine, GFR and BMI were not associated with any one of the groups (Table 2). Moreover, no significant differences were seen with regard to adverse biopsy proven events in the 1st year (Supplementary Table 1) or creatinine and GFR in the first 5 years after transplantation (Supplementary Table 2, Figure 4).

**Table 2:** Comparison of donor parameters and ischemia time in immunohistochemical subgroups of time zero biopsies.

		**Positive**	**Negative**	**IgA**	**FH**	**C3high**	**C3low**	***p*-value association with positive/negative**	***p*-value association with negative/IgA/FH/C3high/C3low**
Donor parameters	Donor male/female	60/47	28/57	14/11	10/3	7/7	24/24	**0.001**	**0.016, negative 0.027**
	Living/deceased	19/88	31/54	8/17	1/12	0/14	10/38	**0.003**	0.013, p.h. n.s.
	Donor age	51 (6/82), 107	54 (1/86), 85	57 (28/82), 25	48.0 (8/66), 13	54.5 (22/79), 14	50 (14/75), 48	0.053	0.08
	Donor creatinine^*^ (mg/dl)	0.89 (0.32/5.63), 98	0.8 (0.28-4.1), 79	0.9 (0.58/1.86), 25	0.96 (0.32/3.92), 9	0.99 (0.50/5.63), 14	0.8 (0.44/4.12), 44	**0.024**	0.059
	Donor GFR (ml/min)^*^	90.2 (13.16/135.13), 98	88.2 (17.51/158.79), 79	88.55 (36.48/119.87), 25	85.56 (19.97/134.86), 9	58.27 (13.16/135.13), 14	92.98 (17.53/118.96), 44	0.465	0.305
	Donor smoker (y/n)	40/62	27/56	6/18	6/6	3/11	22/23	0.347	0.123
	Donor proteinuria (y/n)	29/73	14/67	5/17	4/9	9/5	10/36	0.077	**0.005, C3 high 0.002**
	Donor BMI	26.12 (9.8/65.3), 99	26.3 (15.7/48.4), 81	27.0 (22/44), 25	26.01 (9.8/34.0), 10	25.68 (20.2/63.4), 14	26,2 (20.2/65.3), 44	0.437	0.809
	Donor DM (y/n)	8/89	3/78	1/20	1/11	2/12	2/42	0.21	0.561
	Donor aHT (y/n)	30/68	27/54	5/16	4/8	6/8	12/32	0.697	0.750
Ischemia	Cold (minutes)	664.5 (30/1438), 110	540 (26/1148), 86	492 (44/1438), 28	689 (368/860), 11	700.5 (345/1056), 16	664.5 (30/1192), 48	**0.024**	0.134
	Warm (minutes)	35 (16/121), 111	37 (23/90), 87	36.5 (16/91), 28	30 (19/121), 11	33 (24/55), 16	38 (16/92), 49	0.406	0.693

*Parameters are shown as either median (min/max), total number of analyzed cases or number of yes/no (y/n) cases, if not indicated otherwise*.

* *before organ explantation. For the kidney pairs with the same immune profile only one was included into the statistical analysis of donor parameters. DM, diabetes mellitus; aHT, arterial hypertension; p.h.n.s., post hoc not significant. Data with a gray background show groups with significant differences (indicated in bold) after post-hoc testing*.

C3high and C3low groups were separately analyzed as donor proteinuria was more frequent in donors with C3high deposits than in C3low cases (*p* = 0.003, data not shown).

When comparing all cases with glomerular immune reactivity to the control group, presence of glomerular immune reactivity was significantly associated with deceased and male donors (*p* = 0.003 and 0.001, respectively). Cold ischemia was significantly longer (*p* = 0.024) and donor creatinine higher (*p* = 0.024) in transplants with immune reactivity than in controls (Table 2). No differences were observed with regard to histological findings in time zero and 1-year biopsies, biopsy-proven adverse events, creatinine and GFR in the 5 years after transplantation or other donor parameters (Tables 1, 2, Supplementary Tables 1, 2 and Figure 4).

## Discussion

In our experience, glomerular immune reactivity in time zero biopsies is a frequent finding. To substantiate this observation and to better understand its relevance for future organ function we analyzed a total of 204 time zero biopsies including 161 consecutive cases by immunohistochemistry.

Earlier studies mainly focusing on IgA in time zero biopsies reported frequencies of glomerular IgA-reactivity ranging from 6.9 to 32.1% (1–8). Our findings with 8.7% IgA-reactivity were in the lower range of these earlier reports. This may in part be a consequence of differences in the ethnicities in different cohorts (8). Besides IgA-reactivity we frequently found glomerular C3 (32.9%), fullhouse patterns (1.9%) and mesangial immune-reactivity NOS (1.2%) as well as one case of membranous pattern (0.6%). We decided to separately assess cases with little mesangial C3 reactivity (C3low, 24.2%) and moderate to strong C3 reactivity (C3high, 8.7%), as in the latter donor proteinuria was significantly more common than in the C3low group, which could indicate a different significance of both findings. Interestingly, high levels of C3 (C3high) were only detected in time zero biopsies of deceased donors, whereas mild C3 reactivity was found with similar frequencies in kidneys from living and deceased donors. Proteinuria is an exclusion criterion for living donors, which could explain this finding. Additionally, the alternative complement pathway is activated by spontaneous hydrolysis (12). One could postulate that this mechanism might be augmented during the perimortal phase and transplantation procedure in deceased donors, which might trigger the activation of the complement pathway. The significance of glomerular C3 reactivity remains, however, unclear. It might indicate genuine and relevant complement activation in the donor or just an epiphenomenon of perimortal processes without pathophysiological relevance.

Mesangial reactivity for C3 and also IgM has been observed previously in time zero biopsies. Curschellas et al. reported glomerular C3 and IgM in 18.6 and 65.7%, respectively, which was not interpreted as glomerulonephritis and had no impact on serum creatinine 1 year after transplantation (7). Others reported C3 and IgM in 6.1 and 31.4% of time zero biopsies (3) and 39.4 and 52.6% of donor kidneys (8). In contrast, in a large study of necropsies including subjects, which had committed suicide or died of a violent death, mesangial IgM was present in only 2.5 and C3b in 0.2% (13). The reason why in our time zero biopsies the frequency of C3 deposits was very high may have something to do with the fact that C3 is of limited stability (14) and might get lost especially in the case of necropsies. Moreover, in contrast to the previous studies we applied immunohistochemistry on FFPE-material instead of immunofluorescence on frozen sections, frequently used before (3, 13). Isolated deposits of IgM and/or C1q (found in 79.4 and 14.7%) were not interpreted as a significant finding by us, as some degree of mesangial IgM is observed in the vast majority of renal biopsies [personal observation, (7)]. The molecular sizes of IgM-pentamers and C1q-protein complexes are very large (15, 16), so that both proteins might be prone to trapping in the mesangium. Our observation that mesangial IgM was associated with higher glomerular filtration rates in the donors might support this notion, meaning that with stronger filtration and increased transglomerular flow more protein can be trapped.

In our cohort 13 kidney pairs were included, which were transplanted to different recipients. Interestingly, all but one pair showed the same immune profile in both time zero biopsies. In only one case one kidney showed negative findings whereas the other showed mild mesangial C3 deposits. This concordance in the vast majority of time zero biopsies implicates that the immune-reactivity observed is largely determined by donor parameters rather than by peri-transplantation circumstances. Accordingly, IgA deposits were observed in 4–10.8% (13, 17–19) of unpreselected renal specimens, which also argues that immune reactivity can be found in randomly selected native kidneys and is not a mere epiphenomenon of the transplantation procedure. Moreover, presence of immune reactivity was associated with deceased donor transplants and male gender of the donors, further substantiating the idea that donor parameters are relevant for the observed immune reactivity. The association of immune reactivity with longer cold ischemia may be a consequence of the increased numbers of deceased donors in this group and does not necessarily indicate that cold ischemia is causative for glomerular immune reactivity. Accordingly, in the group of deceased donors the presence or absence of immune reactivity was not significantly associated with cold ischemia (*p* = 0.95, data not shown).

Looking at the time-course of persistence of immune reactivity in follow-up biopsies, a positive result of immunohistochemistry was particularly common early after transplantation and in most cases disappeared in further follow-up biopsies, when available. This disappearance of glomerular immune reactivity over time goes well in line with earlier reports (4, 6–8) and might indicate a wash-out mechanism being operational. In two cases, persistence in more than one biopsy was observed. Intriguingly, in both cases the immune reactivity had an immune profile compatible with a recurrence of the native kidney disease, as reported in the clinical files, so that it was not possible to decide whether a true persistence or a recurrence was observed in the transplant. In fact, it was proposed that latent IgA deposits in time zero biopsies might predispose to IgA-recurrence in the transplant (5).

Immune reactivity in time zero biopsies neither influenced the histomorphological parameter in time zero and 1-year biopsies nor the number of biopsy-proven adverse events in the 1st year after renal transplantation. Moreover, no significant impact on 1 to 5-year follow-up with regard to graft function and survival was observed. Accordingly, no influence on 1-year creatinine was observed by Curschellas et al. (7) and also Sofue et al. did not observe a difference in renal function between cases with and without IgA deposits in time zero biopsies at 1 year (6). Another report stated that cases with mesangial IgA deposition were prone to delayed graft function and development of borderline changes when compared to controls, whereas graft survival at 1 and 3 years was similar in both groups (4). In this cohort, however, only cases with mesangial proliferation and marked IgA deposition where included, mitigating the comparability with our cohort (4).

Limitations of our study include the retrospective nature of the analyses as well as the relatively low numbers of cases included in the different subgroups, which we tried to compensate by extending the cohort. Time zero biopsies were collected before transplantation, therefore, prognostically adverse deposition of complement and immunoglobulins at a later point in time due to ischemia/reperfusion injury cannot be ruled out, as this would have been missed in our analyses. Native kidney disease in most cases with immune reactivity in time zero biopsies was usually not diagnosed in-house, so that one had to rely on the clinically reported diagnoses when evaluating for recurrence of native kidney disease. Moreover, we did not perform light chain immunohistochemistry, so that subtypes of glomerulonephritis with monoclonal immunoglobulin deposition might have been overlooked.

Taken together, we found glomerular immune reactivity to be a very frequent finding in time zero biopsies, which largely seems to be determined by donor parameters, maybe with some enhancement of the complement pathway in deceased donors. In the vast majority of cases this reactivity disappeared after transplantation. It had no impact on graft function or survival, not only in the 1st year as previously reported, but also in the long-run up to 5 years post renal transplantation and did not promote increased scarring of the transplant in 1 year biopsies. Therefore, it appears very justifiable to proceed with the current routine to transplant organs without testing for incidental glomerular immune reactivity before transplantation, as no increased risk of inferior outcome or raised need for closer follow-up or increased immunosuppression appears to be warranted.

## Data Availability Statement

The original contributions presented in the study are included in the article/Supplementary Material, further inquiries can be directed to the corresponding author/s.

## Ethics Statement

The studies involving human participants were reviewed and approved by Ethics Committee of the Friedrich-Alexander-University (Re.-No. 4415). Written informed consent to participate in this study was provided by the participants' legal guardian/next of kin.

## Author Contributions

EV collected and analyzed data, performed experiments, and edited the manuscript. SS collected analyzed data and performed experiments. CD collected and analyzed data and edited the manuscript. KA collected data and edited the manuscript. JS and BB collected clinical data and edited the manuscript. FF contributed to statistical analyses and edited the manuscript. KH and HA collected clinical data. MB-H initiated the study and collected and analyzed data and wrote the manuscript. All authors contributed to the article and approved the submitted version.

## Conflict of Interest

The authors declare that the research was conducted in the absence of any commercial or financial relationships that could be construed as a potential conflict of interest.

## Supplementary Material

Supplementary Figure 1 Ultrastructural findings in persisting immune reactivity in follow-up biopsies. In the left column examples of persisting immune reactivity in follow-up biopsies are shown as indicated. The right column shows the corresponding ultrastructural findings with osmiophilic deposits indicated by arrows, which in the lower panel were very small. All light microscopic pictures were taken at an original magnification of 400x. Scale bars indicate 100 μm. Ultrastructural micrographs all in 5,000x original magnification.

Supplementary Table 1 Comparison of biopsy findings in immunohistochemical subgroups of time zero biopsies.

Supplementary Table 2 Comparison of follow-up parameters in immunohistochemical subgroups of time zero biopsies.

## References

[1] Rosenberg H Martinez P Vaccarezza A Martinez L. [A morphologic study of 103 kidneys donated for renal transplantation. Rev Med Chil. (1989) 117:1344–50. 2519371

[2] Rosenberg HG Martinez PS Vaccarezza AS Martinez LV. Morphological findings in 70 kidneys of living donors for renal transplant. Pathol Res Pract. (1990) 186:619–24. 10.1016/S0344-0338(11)80225-6 2149595

[3] Suzuki K Honda K Tanabe K Toma K Nihei H Yamaguchi Y. Incidence of latent mesangial IgA deposition in renal allograft donors in Japan. Kidney Int. (2003) 63:2286–94. 10.1046/j.1523-1755.63.6s.2.x 12753320

[4] Ji S Liu M Chen J Yin L Sha G Chen H. The fate of glomerular mesangial IgA deposition in the donated kidney after allograft transplantation. Clin Transplant. (2004) 18:536–40. 10.1111/j.1399-0012.2004.00206.x 15344956

[5] Moriyama T Nitta K Suzuki K Honda K Horita S Uchida K. Latent IgA deposition from donor kidney is the major risk factor for recurrent IgA nephropathy in renal transplantation. Clin Transplant. (2005) 19(Suppl. 14):41–8. 10.1111/j.1399-0012.2005.00403.x 15955168

[6] Sofue T Inui M Hara T Moritoki M Nishioka S Nishijima Y. Latent IgA deposition from donor kidneys does not affect transplant prognosis, irrespective of mesangial expansion. Clin Transplant. (2013) 27 (Suppl. 26):14–21. 10.1111/ctr.12158 24299231

[7] Curschellas E Landmann J Durig M Huser B Kyo M Basler V. Morphologic findings in “zero-hour” biopsies of renal transplants. Clin Nephrol. (1991) 36:215–22. 1752070

[8] Gaber LW Khan FN Graviss EA Nguyen DT Moore LW Truong LD. Prevalence, characteristics, and outcomes of incidental IgA glomerular deposits in donor kidneys. Kidney Int Rep. (2020) 5:1914–24. 10.1016/j.ekir.2020.08.018 33163712 PMC7609995

[9] Levey AS Stevens LA Schmid CH Zhang YL Castro AF Feldman HI. A new equation to estimate glomerular filtration rate. Ann Int Med. (2009) 150:604–12. 10.7326/0003-4819-150-9-200905050-00006 19414839 PMC2763564

[10] Roufosse C Simmonds N Clahsen-van Groningen M Haas M Henriksen KJ Horsfield C. A 2018 reference guide to the banff classification of renal allograft pathology. Transplantation. (2018) 102:1795–14. 10.1097/TP.0000000000002366 30028786 PMC7597974

[11] Beasley TM Schumacker RE. Multiple regression approach to analyzing contingency tables: post hoc and planned comparison procedures. J Exp Educ. (1995) 64:79–93. 10.1080/00220973.1995.9943797

[12] Thurman JM Nester CM. All things complement. Clin J Am Soc Nephrol. (2016) 11:1856–66. 10.2215/CJN.01710216 27340286 PMC5053787

[13] Varis J Rantala I Pasternack A Oksa H Jantti M Paunu ES. Immunoglobulin and complement deposition in glomeruli of 756 subjects who had committed suicide or met with a violent death. J Clin Pathol. (1993) 46:607–10. 10.1136/jcp.46.7.607 8157744 PMC501386

[14] Larsen S. Glomerular immune deposits in kidneys from patients with no clinical or light microscopic evidence of glomerulonephritis. Assessment of the influence of autolysis on identification of immunoglobulins and complement. Acta Pathol Microbiol Scand A. (1979) 87:313–9. 10.1111/j.1699-0463.1979.tb00058.x 393070

[15] Roberts-Thomson PJ Shepherd K. Molecular size heterogeneity of immunoglobulins in health and disease. Clin Exp Immunol. (1990) 79:328–34. 10.1111/j.1365-2249.1990.tb08091.x 2317941 PMC1534949

[16] Mii A Shimizu A Masuda Y Fujita E Aki K Ishizaki M. Current status and issues of C1q nephropathy. Clin Exp Nephrol. (2009) 13:263–74. 10.1007/s10157-009-0159-5 19373520

[17] Waldherr R Rambausek M Duncker WD Ritz E. Frequency of mesangial IgA deposits in a non-selected autopsy series. Nephrol Dial Transplant. (1989) 4:943–6. 10.1093/ndt/4.11.943 2516884

[18] Sinniah R. Occurrence of mesangial IgA and IgM deposits in a control necropsy population. J Clin Pathol. (1983) 36:276–9. 10.1136/jcp.36.3.276 6338054 PMC498197

[19] Varis J Rantala I Pasternack A. Immunofluorescence of immunoglobulins and complement in kidneys taken at necropsy. J Clin Pathol. (1989) 42:1211–4. 10.1136/jcp.42.11.1211 2685055 PMC501984

