# Supplementary figures and images for: Significance of Glomerular Immune Reactivity in Time Zero Biopsies for Allograft Survival Beyond IgA
Source: Front Med (Lausanne). 2021 Apr 6;8:656840. doi: 10.3389/fmed.2021.656840 (PMC8057301; doi:10.3389/fmed.2021.656840)

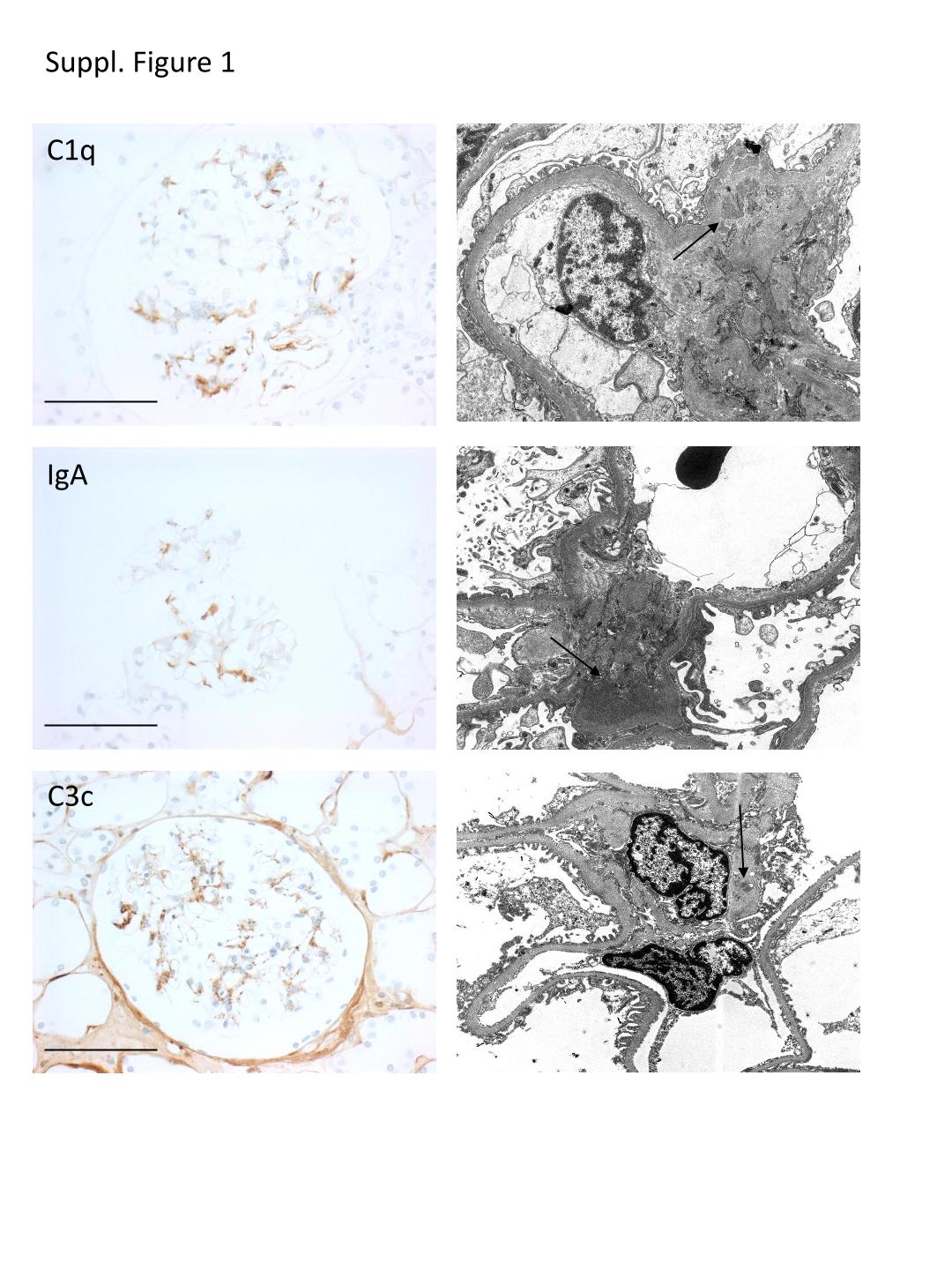

Supplement: Supplementary Figure 1 — Ultrastructural findings in persisting immune reactivity in follow-up biopsies. In the left column examples of persisting immune reactivity in follow-up biopsies are shown as indicated. The right column shows the corresponding ultrastructural findings with osmiophilic deposits indicated by arrows, which in the lower panel were very small. All light microscopic pictures were taken at an original magnification of 400x. Scale bars indicate 100 μm. Ultrastructural micrographs all in 5,000x original magnification. [file Image_1.JPEG]
