# Supplementary material for: Significance of Glomerular Immune Reactivity in Time Zero Biopsies for Allograft Survival Beyond IgA
Source: Front Med (Lausanne). 2021 Apr 6;8:656840. doi: 10.3389/fmed.2021.656840 (PMC8057301; doi:10.3389/fmed.2021.656840)
Supplement: Supplementary Table 1 — Comparison of biopsy findings in immunohistochemical subgroups of time zero biopsies. [file Data_Sheet_1.docx]

**Supplementary Table 1: Comparison of biopsy findings in immunohistochemical subgroups of time zero biopsies**

|  |  |  | **positive** | **negative** | **IgA** | **FH** | **C3high** | **C3low** | **p-value**  **association with**  **positive/**  **negative** | **p-value**  **association with**  **negative/IgA/FH/ C3high/C3low** |
| --- | --- | --- | --- | --- | --- | --- | --- | --- | --- | --- |
| **Biospy findings** | **time zero biopsies** | glomeruli (n) | 10  (2/29), 115 | 10  (1/23), 88 | 11  (5/24), 28 | 11.5  (6/21), 14 | 9  (2/14), 16 | 10  (4/20), 50 | n.a. | n.a. |
|  |  | GS % | 0  (0/82), 116 | 6.97  (0/80), 88 | 0  (0/20), 28 | 10.8  (0/46), 14 | 10  (0/82), 17 | 0  (0/43), 50 | 0.405 | 0.53 |
|  |  | MM (y/n) | 51/65 | 38/50 | 13/15 | 4/10 | 4/13 | 22/28 | 0.911 | 0.123 |
|  |  | IFTA % | 5  (0/95), 116 | 0  (0/70), 88 | 5  (0/20), 28 | 5  (0/20), 14 | 0  (0/95), 17 | 5  (0/40), 50 | 0.088 | 0.282 |
|  |  | AS (score) | 1  (0/3), 116 | 1  (0/3), 88 | 1  (0/3), 28 | 0  (0/1), 14 | 1  (0/3), 17 | 1  (0/3), 50 | 0.268 | 0.5 |
|  |  | ATI (score) | 4  (1/4), 114 | 4  (1/4), 88 | 4  (2/4), 27 | 4  (3/4), 14 | 4  (2/4), 16 | 4  (1/4), 50 | 0.266 | 0.479 |
|  | **1year biopsies** | glomeruli (n) | 12  (3/24), 61 | 10  (3/32), 50 | 10.5  (4/24), 16 | 9  (3/16), 5 | 12  (8/22), 7 | 12  (3/24), 29 | n.a. | n.a. |
|  |  | GS % | 0  (0/57.1), 61 | 4.6  (0/95), 50 | 5.4  (0/29), 16 | 22.2  (0/57), 5 | 5  (0/25), 7 | 0  (0/27), 29 | 0.305 | 0.183 |
|  |  | MM (y/n) | 15/46 | 15/35 | 5/11 | 2/3 | 4/3 | 4/25 | 0.523 | 0.172 |
|  |  | IFTA % | 10  (0/60), 60 | 12.5  (0/100), 50 | 10  (10/40), 16 | 25  (15/60), 5 | 20  (5/30), 7 | 10  (5/35), 28 | 0.993 | 0.16 |
|  |  | AS (score) | 1 (0/3),  59 | 1 (0/3),  50 | 1 (0/3),  15 | 1 (0/2),  5 | 1 (0/2),  6 | 1 (0/2),  29 | 0.777 | 0.992 |
|  | **Follow-up biopsies in the 1st year** | No. of biopsies (n) | 2  (0/8), 116 | 2  (0/9), 88 | 1  (0/8), 28 | 1  (0/4), 14 | 2  (0/4), 17 | 2  (0/5), 50 | 0.216 | 0.478 |
|  |  | adverse events bx (n) | 0.5  (0/5), 92 | 1  (0/7), 77 | 1  (0/4), 21 | 0  (0/2), 11 | 0  (0/3), 15 | 1  (0/5), 39 | 0.775 | 0.79 |

Parameters are shown as median (min/max), number of analyzed cases or yes/no (y/n) if not indicated otherwise. GS % = percentage of global glomerulosclerosis, MM= mesangial matrix expansion, IFTA= interstitial fibrosis and tubular atrophy, AS= arteriosclerosis, ATI= acute tubular injury, adverse events bx= number of biopsies within the 1^st^ year post transplantation with signs of rejection (Borderline, antibody or T-cell mediated) or polyomavirus nephropathy; n.a.= not assessed

**Supplementary Table 2: Comparison of follow-up parameters in immunohistochemical subgroups of time zero biopsies**

|  |  | **positive** | **negative** | **IgA** | **FH** | **C3high** | **C3low** | **p-value association with**  **positive/**  **negative** | **p-value association with**  **negative/IgA/ FH/**  **C3high/C3low** |
| --- | --- | --- | --- | --- | --- | --- | --- | --- | --- |
| **Follow-up** | HD post-Rtx (n) | 0  (0/9),  106 | 0  (0/4),  85 | 0  (0/7),  26 | 0  (0/6),  14 | 0  (0/3),  14 | 0  (0/9),  45 | 0.440 | 0.8 |
|  | Rtx-survival (y/n) | 108/7 | 84/4 | 26/2 | 14/0 | 15/2 | 47/2 | 0.76 | 0.607 |
|  | Patient survival (y/n) | 113/2 | 87/1 | 26/2 | 14/0 | 17/0 | 49/1 | 1.0 | 0.126 |
|  | Primary /delayed/no function, organ loss or death | 94/15/5 | 76/11/1 | 24/2/2 | 11/3/0 | 12/3/2 | 40/7/1 | 0.392 | 0.290 |
|  | GFR 1 year (ml/min) | 52.0  (8/105),  101 | 51.8  (21/111),  78 | 48.2  (22/93),  25 | 53.8  (21/92),  12 | 45.3  (28/91),  15 | 57.3  (29/105),  44 | 0.760 | 0.450 |
|  | GFR 2 years (ml/min) | 48.3  (8.2/124.9),  71 | 49.2 (16.9/124.2), 52 | 43.4  (8.2/87.5),  19 | 50.7  (20.9/107),  9 | 62.8  (33.5/78),  9 | 57.1  (17/124.9),  30 | 0.563 | 0.203 |
|  | GFR 3 years (ml/min) | 51.4  (7.2/116),  61 | 50.1  (18.2/108),  49 | 41.0  (23.7/102.3),  18 | 64.7  (33.6/104.3),  7 | 58.9  (27.3/76),  8 | 53.6  (7.2/116),  23 | 0.815 | 0.264 |
|  | GFR 4 years (ml/min) | 47.2  (15.5/137.9),  42 | 49.5  (19.2/108),  37 | 40.3  (18.9/83.1),  17 | 52.8  (15.5/137.9),  7 | 61.4  (27.9/78.8),  8 | 51.5(25.8/105.6),  8 | 0.589 | 0.338 |
|  | GFR 5 years (ml/min) | 46.9 (11.3/122.4), 28 | 53.9 (20.4/114.7), 36 | 46.3  (29.7/83.1),  13 | 48.6  (11.3/122.4),  8 | 67.6  (19.1/74.6),  7 | / | 0.330 | 0.320 |

Parameters are shown as median (min/max), number of analyzed cases or yes/no (y/n) if not indicated otherwise.

HD= hemodialysis, Rtx= renal transplant, GFR= glomerular filtration rate
